# Property in Prescriptions

**Published:** 1872-10

**Authors:** 


### PROPERTY IN PRESCRIPTIONS.

As bearing upon a recent discussion in our columns, we insert the following editorial from the *Philadelphia Medical Times*, of October 5th, 1872:

"A question has recently been raised afresh as to who has the right to a physician's prescription. Does it belong to the writer, to the patient who pays for it, or to the druggist who puts it on file after dispensing the medicine in accordance with it? It seems to us that the matter is easily settled by common sense. A physician, having examined a patient, writes directions for the putting up of the remedies he considers suitable under the circumstances; the apothecary follows his instructions, without responsibility except to conform to them; the patient takes the label as his guide, and does as he has been advised. Supposing all this to be done, the physician evidently has no more authority over that prescription, since it is the apothecary's voucher for his preparation of the medicine, and, in case of anything going wrong, would be his only safeguard; the latter has none, for it was merely an instruction to him to compound certain drugs; the patient can have none, for the circumstances under which it was given, and which he is not supposed to appreciate, may have wholly changed before six hours have elapsed from the writing of it.

"The practice in London, and perhaps all over England, and on the Continent for aught we know, is for the druggist to make and retain a copy of the prescription, returning the original to the patient.

"We are well aware that it is the custom here for patients to send back their phials for renewal, and for druggists to refill them, without the sanction of the physician whose name is appended to prescription No. 12,345, or whatever it may be, being obtained. But this is done at their own risk; and should salivation, narcotic or irritant poisoning, or any other untoward result, ensue upon the taking of medicine so renewed, the physician's skirts would be clear. It is also the custom, and a very bad one, for patients to say to their friends: 'Dr. So-and-so gave me a prescription which I am sure would do you good; I will give you the number of it.' We have known half a dozen persons to obtain medicine in this way, without any certainty that their cases were analogous to the one for which the remedies were ordered.

"Sometimes, to save trouble, physicians who have occasion to make frequent prescriptions of the same compounds, will give numbered formulæ to certain druggists, and order them by numbers or by special designations. But this does not change the principle. If a surgeon examines a case of disease of the eye, and writes a prescription for 'collyrium adstringens,' or for 'tonic No. 3,' any apothecary who has the corresponding formulæ can follow that direction, as if the ordinary terms were used. It is merely an instruction to him to furnish certain remedies adapted to the existing state of the patient.

"This is the great principle for which we contend: that a prescription given in any case is not a thing sold and made over either to patient or to druggist, but a direction supposed to be based on that understanding of the morbid conditions which the physician alone has; and since these conditions may or may not exist at a later stage or in another case, it is at the druggist's risk that he recommends, or at the patient's risk that he orders, the filling of any prescription without direct medical sanction."

